# Overexpression of Adenylyl Cyclase Encoded by the *Mycobacterium tuberculosis* Rv2212 Gene Confers Improved Fitness, Accelerated Recovery from Dormancy and Enhanced Virulence in Mice

**DOI:** 10.3389/fcimb.2017.00370

**Published:** 2017-08-17

**Authors:** Margarita O. Shleeva, Tatyana K. Kondratieva, Galina R. Demina, Elvira I. Rubakova, Anna V. Goncharenko, Alexander S. Apt, Arseny S. Kaprelyants

**Affiliations:** ^1^Federal Research Centre ‘Fundamentals of Biotechnology’ of the Russian Academy of Sciences, A. N. Bach Institute of Biochemistry Moscow, Russia; ^2^Department of Immunology, Laboratory for Immunogenetics, Central Institute for Tuberculosis Moscow, Russia; ^3^Department of Immunology, School of Biology, Lomonosov Moscow State University Moscow, Russia

**Keywords:** *Mycobacterium tuberculosis*, cAMP, adenylyl cyclase, Rv2212, dormant mycobacteria

## Abstract

Earlier we demonstrated that the adenylyl cyclase (AC) encoded by the *MSMEG_4279* gene plays a key role in the resuscitation and growth of dormant *Mycobacterium smegmatis* and that overexpression of this gene leads to an increase in intracellular cAMP concentration and prevents the transition of *M. smegmatis* from active growth to dormancy in an extended stationary phase accompanied by medium acidification. We surmised that the homologous *Rv2212* gene of *M. tuberculosis* (*Mtb*), the main cAMP producer, plays similar physiological roles by supporting, under these conditions, the active state and reactivation of dormant bacteria. To test this hypothesis, we established *Mtb* strain overexpressing *Rv2212* and compared its *in vitro* and *in vivo* growth characteristics with a control strain. *In vitro*, the AC-overexpressing pMind*Rv2212* strain demonstrated faster growth in a liquid medium, prolonged capacity to form CFUs and a significant delay or even prevention of transition toward dormancy. AC-overexpressing cells exhibited easier recovery from dormancy. *In vivo*, AC-overexpressing bacteria demonstrated significantly higher growth rates (virulence) in the lungs and spleens of infected mice compared to the control strain, and, unlike the latter, killed mice in the TB-resistant strain before month 8 of infection. Even in the absence of selecting hygromycin B, all pMind*Rv2212* CFUs retained the *Rv2212* insert during *in vivo* growth, strongly suggesting that AC overexpression is beneficial for bacteria. Taken together, our results indicate that cAMP supports the maintenance of *Mtb* cells vitality under unfavorable conditions *in vitro* and their virulence *in vivo*.

## Introduction

Asymptomatic forms of tuberculosis (TB), when infected individuals carry the pathogen in dormant state without notable symptoms of the disease for a very long time, are termed latent tuberculosis infection (LTBI). In some of these latently infected individuals, infection eventually transits to the active state, becomes contagious and seriously affects epidemiological situation (Barry et al., 2009). Conditions under which dormant *Mycobacterium tuberculosis* (*Mtb*) transits to active multiplication (resuscitates), as well as the mechanisms of transition, are poorly understood. Thus, currently the problem of LTBI development and reactivation is one of the most important in infectious medicine.

In studies aimed at understanding the molecular mechanisms underlying Mtb dormancy and resuscitation, a significant amount of information has been obtained using experimental models *in vitro* which mimic the development of mycobacterial dormancy and resuscitation (Shleeva et al., 2002, 2011). For instance, the role of the adenylyl cyclase (AC) encoded by the *MSMEG_4279* gene has been elucidated through a model of dormant *Mycobacterium smegmatis* resuscitation. It was shown that the *M. smegmatis* strain carrying the knock-out mutation in the *MSMEG_4279* gene was unable to resuscitate and required the addition of exogenous cAMP for reactivation. Moreover, *M. smegmatis* and *M. tuberculosis* transformation with the plasmid containing the *MSMEG_4279* gene expressed under the Tet-promoter, which led to its hyper-expression and an increase in intracellular cAMP concentration, prevented the transition of the two species of bacteria to dormancy under stressful conditions (Shleeva et al., 2013). This suggests that in the presence of high amounts of cAMP provided by the AC the bacteria retained their active state. However, direct evidence for the “anti-dormant” role of the *M. tuberculosis* gene(s) encoding AC for *Mtb* remained lacking.

The homolog of *MSMEG_4279* in *Mtb* is the *Rv2212* gene, which is the major producer of cAMP among 16 biochemically active AC-encoding genes present in the genome (Abdel Motaal et al., 2006; Knapp and McDonough, 2014). We assumed that under stressful conditions the *Rv2212* gene, like *MSMEG_4279*, contributes to the retention of the active metabolic state of *Mtb*, as well as the reactivation of dormant bacteria due to an increase in cAMP production. To test this hypothesis, we established a novel *Mtb* strain carrying the plasmid containing *Rv2212* expressed under the Tet-promoter and compared the *in vitro* and *in vivo* phenotypes of this *Rv2212*-overexpressing strain with the control strain carrying the “empty” plasmid. Our data clearly demonstrate that the *Rv2212*-dependent AC activity, which results in elevated cAMP production, supports *Mtb* vitality under unfavorable conditions in a culture medium and inside the host, as well as its resuscitation from dormant state.

## Materials and methods

### Bacterial strains, growth media, and culture conditions

The wild type *Mtb* strain H37Rv and its derivatives carrying plasmids pMind*Rv2212* (see below) and pMind (empty plasmid control) were used. Hygromycin B was added to the growth media at the 50 μg/ml concentration for the plasmid-containing strains. All strains were routinely maintained on the standard Sauton's medium: 0.5 g KH_2_PO_4_, 1.4 g MgSO_4_·7H_2_O, 4 g L-asparagine, 60 ml glycerol; 0.05 g ferric ammonium citrate; 2 g sodium citrate, 0.1 ml 1% ZnSO_4_·7H_2_O, adjusted to l L with H_2_O at pH = 7.0 (adjusted with 1 M NaOH) and supplemented with ADC (albumen, glucose and NaCl) with 0.05% Tween-80 (Connell, 1994).

Mycobacterial populations consisting of bacilli that lost capacity to grow on solid media (“non-culturability,” NC) due to gradual acidification of medium during stationary growth phase were developed as described earlier (Shleeva et al., 2011). Briefly, bacteria were kept for 12–15 days in 50 ml of Sauton's medium supplemented with 0.05% Tween-80 and ADC in 150 ml conical flasks on an orbital shaker (200 rpm). These bacterial cultures served for inoculation of modified Sauton's medium aliquots for establishing NC bacteria. The modified Sauton's medium in which Tween-80 is replaced with 0.025% tyloxapol and ADC—with 0.5% bovine albumin, Cohn-Analog (Sigma) compared to the standard Sauton's medium is hereafter termed “glycerol” medium. Another formulation of the modified Sauton's medium containing 4% glucose and 0.2% glycerol is hereafter termed “glucose”medium. Initial pH values in both modified media were 6.2, contrary to pH = 7.0 in the standard Sauton's medium. An 1-ml inoculum of initial culture was added to 200 ml of modified Sauton's medium put into a 500-ml conical flasks (3–5 flasks per experiment), and incubated with shaking at 37°C for 40–60 days. The pH value of medium was measured periodically, and, when reached 6.0–6.2, the cultures were transferred to capped plastic 50-ml tubes, and 2-(N-Morpholino)ethanesulfonic acid (MES) was added at the final 20 mM concentration to prevent further acidification during long-term storage. Incubation was continued under static conditions (i.e., without agitation) at the room temperature for up to 200 days post-inoculation, in the dark. Periodically, samples were collected from different tubes for estimation of CFU counts and the results provided by the most probable numbers (MPN) assay (see below for details) to monitor appearance of NC bacteria. In some experiments, tetracycline hydrochloride was added to medium at the final concentration 20 ng per ml.

Resuscitation of NC bacteria was performed in reactivation medium—the 1:1 mixture of the standard Sauton's medium and the Nutrient Broth E (NBE) medium (HiMedia, India) supplemented with 0.025% tyloxapol (Sigma) in two formats. For the MPN format, 15 ml plastic test tubes (Corning, USA) containing 2 ml reactivation medium were used. Serially diluted NC bacteria were incubated in triplicates at 37°C for 30–50 days without shaking, and the number of tubes with visible bacterial growth was scored. The MPN values were determined using standard statistical tables (de Man, 1975). For the batch format, dormant *Mtb* bacteria prepared as described above were washed 10 times with PBS, re-suspended in 200 ml of reactivation medium at the initial OD_600_ = 0.2–0.3, put into a 500-ml flask and incubated at 37°C for 45 days with 100–120 rpm agitation. OD values were periodically in cultural samples.

### Estimation of bacterial capacity to form CFU

Bacterial suspensions were serially diluted in fresh Sauton's medium, and triplicate 100-μl samples from each dilution were dropped on Sauton agar. Plates were incubated at 37°C for 21 day, and the numbers of colony forming units (CFU) were counted. The limit of detection was 10 CFU/ml.

### DNA manipulations

The pMind*Rv2212* overexpression plasmid was constructed as follows:

Amplification of genomic *M. tuberculosis* DNA was performed using the primers:

Up2212 5′CTGGATCCTCGCTCACGGCGTCCCACCCTA3′,

Low2212 5′CAACTAGTTCGCGACGGCGACGGAGGGGGATAG3′.

The restriction sites are underlined. Amplification products were firstly cloned into pGEM-T vector (Promega), and then sub-cloned into pMind vector using BamHI and SpeI restriction enzymes (Thermo Scientific). *Escherichia coli* and *M. tuberculosis* were transformed with the pMind*Rv2212* vector by electroporation. After growing in selective medium, colonies were checked using PCR, plasmids from colonies were extracted and checked by sequencing.

### *Ex vivo* CFU analysis

Mycobacterial colonies (*N* = 50) obtained from lung homogenates were analyzed by PCR in order to confirm the presence of the *Rv2212* gene insertion. Amplification was performed using p-Mind specific primers:

Up-pMind 5′CCGGGCCCCGAGCAACACG3′

Low-pMind 5′CCGCAGGCTCGCGTAGGAATCATC3′

All resulting products were about 1,300 bp length, which corresponds to the gene insertion size.

### cAMP determination

*Mtb* samples from different strains and time points of storage were centrifuged at 13,000 g for 5 min. The pellets were treated with 1 ml of 95°C-heated 0.1N HCl for 10 min and immediately frozen. Samples were disrupted in the bead homogenizer FastPrep-24 (MP Biomedicals, The Netherlands), bacterial debris was removed by centrifugation, and cAMP levels were measured in lysates by ELISA, using rabbit anti-cAMP antibodies (1:5,000) and cAMP-peroxidase conjugate (HRP, 1: 20,000) (GenScript, USA). The results were registered in triplicates at 450 nm using Zenyth 3100 microplate reader (Anthos Labtec Instruments, Austria). At least three independent measurements were performed.

### Mice and infection

Mice of inbred strains I/StSnEgYCit (I/St) and C57BL/6JCit (B6) were bred and maintained under conventional, non-SPF conditions at the Animal Facilities of the Central Institute for Tuberculosis (CIT, Moscow, Russia) in accordance with guidelines from the Russian Ministry of Health # 755, and under the NIH Office of Laboratory Animal Welfare (OLAW) Assurance #A5502-11. Water and food were provided *ad libitum*. Female mice of 8–12 week of age at the beginning of experiments were used. All experimental procedures were approved by the CIT animal care committee.

Mice were infected intravenously with 5 × 10^6^ bacteria per mouse in 0.5 ml of sterile PBS. Neither tetracycline nor hygromycin B were administrated in these experiments. To assess mycobacterial loads in spleens and lungs, 0.2 ml of serial 10-fold dilutions of individual whole-organ homogenates obtained from 4 mice per group were plated onto Dubos agar, and colonies were counted after 21–23 days of incubation at 37°C. Two independent experiments were performed and their results combined. Mortality was checked weekly.

### Statistics

OD, CFU, cAMP concentrations, Resuscitation Index (RI) were expressed as mean ± SEM. MPN were calculated used (95%) confidence limits. Significance of the differences for the *in vivo* experiments were estimated by ANOVA, *P* < 0.05 was considered significant.

## Results

### Growth of *Mtb* strains and resuscitation of dormant bacteria *in vitro*

**Figure 1:**
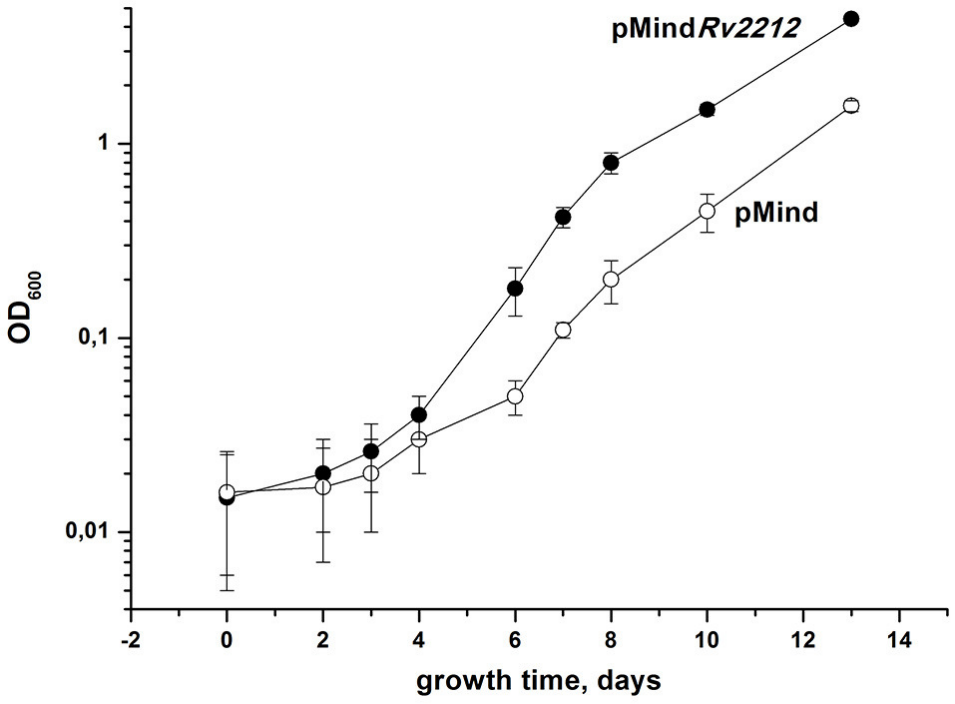
Growth of *M. tuberculosis* strains in the standard Sauton's medium. Recombinant *M. tuberculosis* strains containing pMind*Rv2212* vector (closed circles) or empty pMind vector (open circles) were cultured in the standard Sauton's medium with agitation (200 rpm) at 37°C. Initial size of inoculum was 10^2^ cells per ml. This experiment was repeated three times with similar results. One representative experiment is shown.

To study the influence of AC on *Mtb* growth and resuscitation, we constructed the strain (pMind*Rv2212*) overexpressing AC under the control of the tetracycline (Tet) promoter and demonstrated that this strain displayed a statistically significant increase in the intracellular cAMP levels in exponential phase compared to the empty-vector control strain pMind. The uninduced strain also displayed an elevated level of cAMP expression comparable to that of the induced strain (Figure S1). Apparently, in the absence of the inducer, the background expression level under the Tet promoter in *Mtb* is sufficient, which is in full agreement with our finding in *M. smegmatis* for the overexpressed MSMEG_4279 (a homolog of *Rv2212*) (Shleeva et al., 2013). Thereafter, we compared the growth of these strains in liquid Sauton medium. As shown in Figure 1, the bacteria overexpressing AC grew faster than the control strain. Remarkably, a strong difference in growth rates between the two strains was readily revealed if the starting inoculum contained a low number of bacteria (10^2^ per ml), i.e., provided more unfavorable conditions for growth initiation than larger starting populations did (Mukamolova et al., 2002). Indeed, when a 3-log larger inoculum initiated the growth of the two strains, the speed of multiplication appeared to be almost identical (Figure S2). We also noticed that, in contrast to pMind bacteria, pMind*Rv2212* quickly recovered from an old inoculum and from dried samples (data not shown).

**Figure 2:**
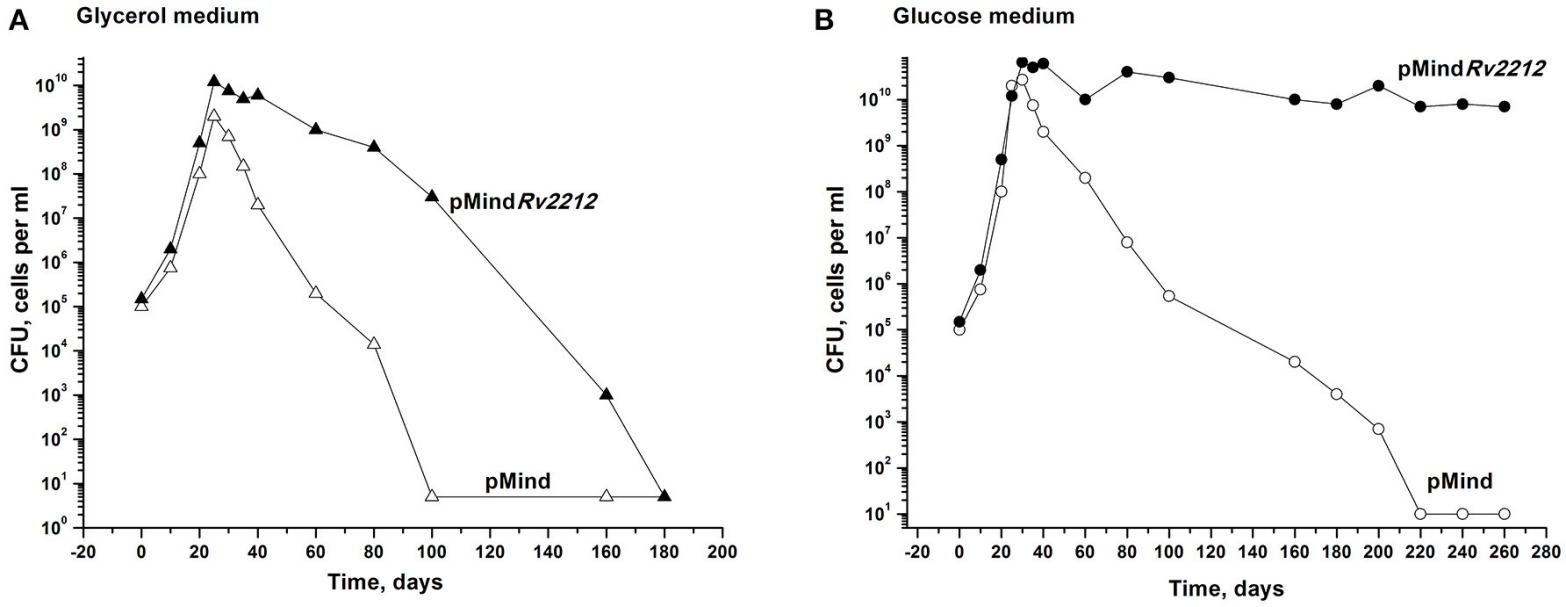
Overexpression of *Rv2212* influences transition of *M. tuberculosis* to dormant “non-culturable” state. Recombinant *Mtb* strains containing pMind*Rv2212* vector (closed circles) or empty pMind vector (open circles) were grown in “glycerol” **(A)** or “glucose” **(B)** modified Sauton's media with agitation (200 rpm) at 37°C. For details see Materials and Methods. Minimum threshold for CFU determination was 10 cells per ml. SEM for the CFU determination was <20%. Experiments shown in **(A)** and in **(B)** were repeated two times and four times, respectively. Typical results are shown.

Next, we studied whether the overexpression of the *Rv2212* gene influences the transition of actively growing bacteria to dormancy. We explored an earlier developed model which makes it possible to accumulate dormant ovoid cells under gradual self-acidification of the growth medium during prolonged stationary phase. These dormant bacteria, which display a distinct morphology and are characterized by “non-culturability” (NC), i.e., a transient loss of capacity to grow on solid media, can be developed in modified Sauton medium, containing either glycerol (Shleeva et al., 2011) or glucose as the major source of carbon. As shown in Figure 2A, in the glycerol-based medium the development of NC pMind*Rv2212* bacteria was significantly postponed compared to NC pMind bacteria. After their development in the glucose-based medium, pMind*Rv2212* bacteria provided identical CFU counts on the solid medium throughout the observation period, whereas the control strain gradually acquired the NC phenotype, although much slower than in the glycerol-based medium (Figure 2B). The difference between two media could be due to faster acidification of the glycerol-based medium during pMind strain growth and the lack of acidification of the glucose-based medium during pMindRv2212 strain growth (data not shown).

**Figure 3:**
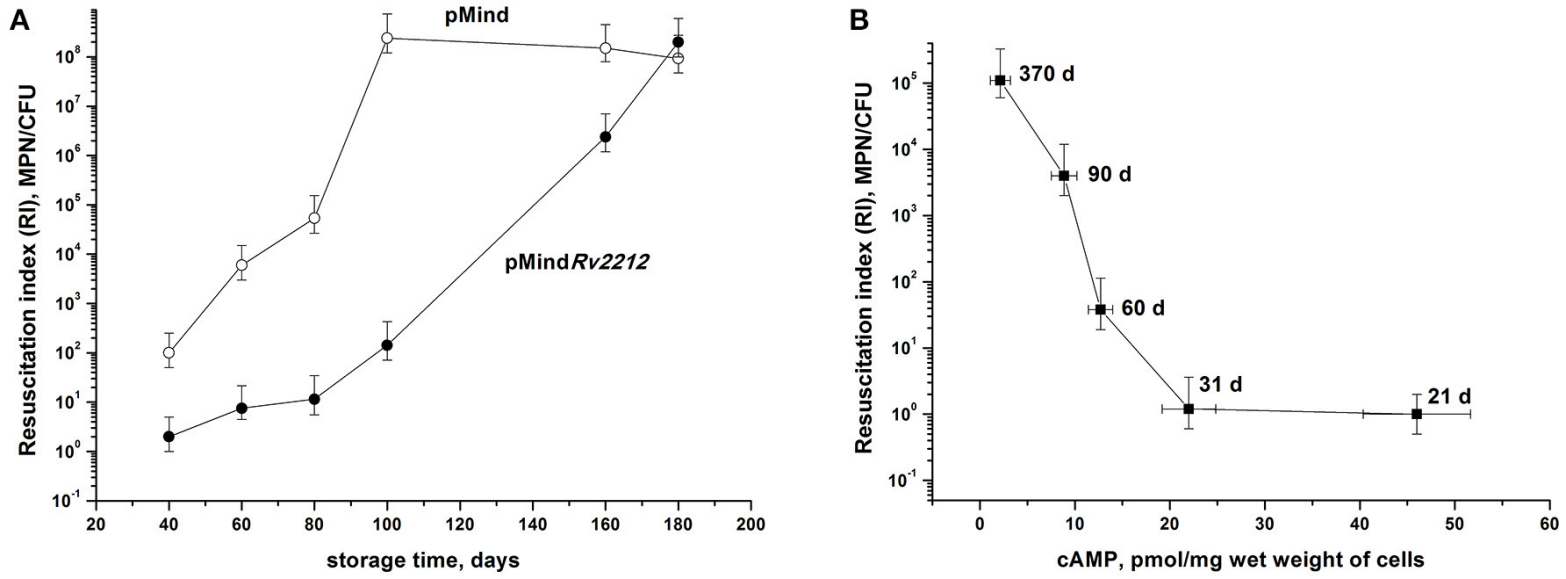
Development of “non-culturability” **(A)** and cAMP content **(B)** of *M. tuberculosis* during dormant bacteria storage. **(A)** *M. tuberculosis* pMind*Rv2212* (closed circles) or pMind (open circles) developed in the “glycerol” modified Sauton's medium were kept statically at the room temperature. Periodically, samples were collected for RI estimation. **(B)** The wild type mycobacteria were grown and kept under identical conditions for monitoring cAMP intracellular concentrations and RI estimation. Sampling days are indicated. Experiment shown in **(A)** was repeated twice, one representative experiment is shown. Results shown in **(B)** display the average from three independent experiments.

At least some NC bacteria are able to resuscitate upon cultivation in an appropriate liquid medium. During culture storage, the numbers of bacteria temporarily existing in the NC state but retaining capacity for resuscitation were assessed using the most probable number (MPN) assay based upon serial dilutions of bacteria incubated in a liquid medium (de Man, 1975; Shleeva et al., 2002). By plotting the MPN assay results against the numbers of CFU (the so-called Resuscitation Index, RI) we evaluated the development of the NC state in the two bacterial strains, as well as the contribution of those dormant bacteria, which retained their resuscitation capacity during long storage, to the total size of bacterial population. As shown in Figure 3A, RI varied between 10^0^ (1, corresponds to fully active, multiplying bacteria) and 10^8^ (almost the entire population consists of NC bacteria). Remarkably, pMind bacteria achieved the highest RI value by day 100 of storage, whereas in pMind*Rv2212* population this condition was achieved not earlier than day 180, indicating significance of the *Rv2212* expression for the maintenance of active growth under long storage. In addition, as shown for the wild type bacteria, there was a clear reverse correlation between the RI value and the content of cAMP in bacterial cells, i.e., gradual increase of bacterial population reaching the NC state during prolonged storage (Figure 3B).

**Figure 4:**
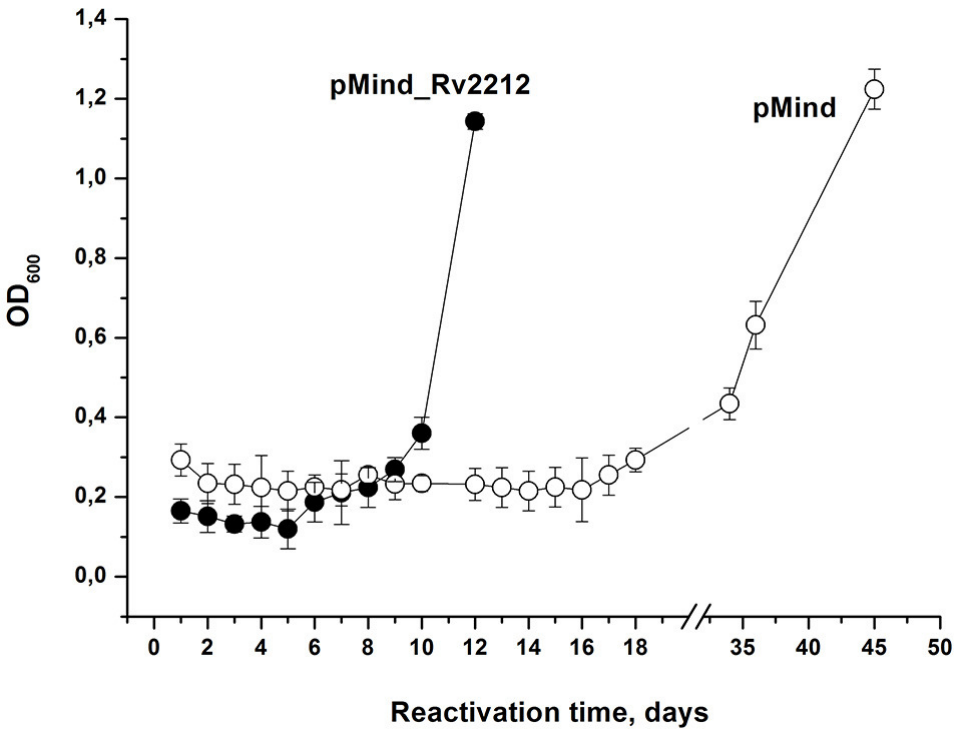
Overexpression of *Rv2212* results in faster resuscitation of dormant *M. tuberculosis*. Non-culturable *Mtb* pMind*Rv2212* (closed circles) or pMind (open circles) obtained from the “glycerol” Sauton's modified medium after 180-d incubation (see the legend to Figure 2A) were washed, inoculated in the reactivation medium and incubated with agitation (100 rpm) at 37°C. For the details see Materials and Methods. The experiment was repeated two times with similar results.

The resuscitation of NC bacteria developed in the glycerol-based medium (see Figure 2A, 180 days) was also assessed using the batch format (Shleeva et al., 2013), when identical numbers of bacteria are inoculated in flasks containing resuscitation medium. In this experiment, pMind*Rv2212* bacteria exhibited significantly shorter lag phase and quicker resuscitation compared to pMind strain (Figure 4).

### Infection caused by two mycobacterial strains in mice

**Figure 5:**
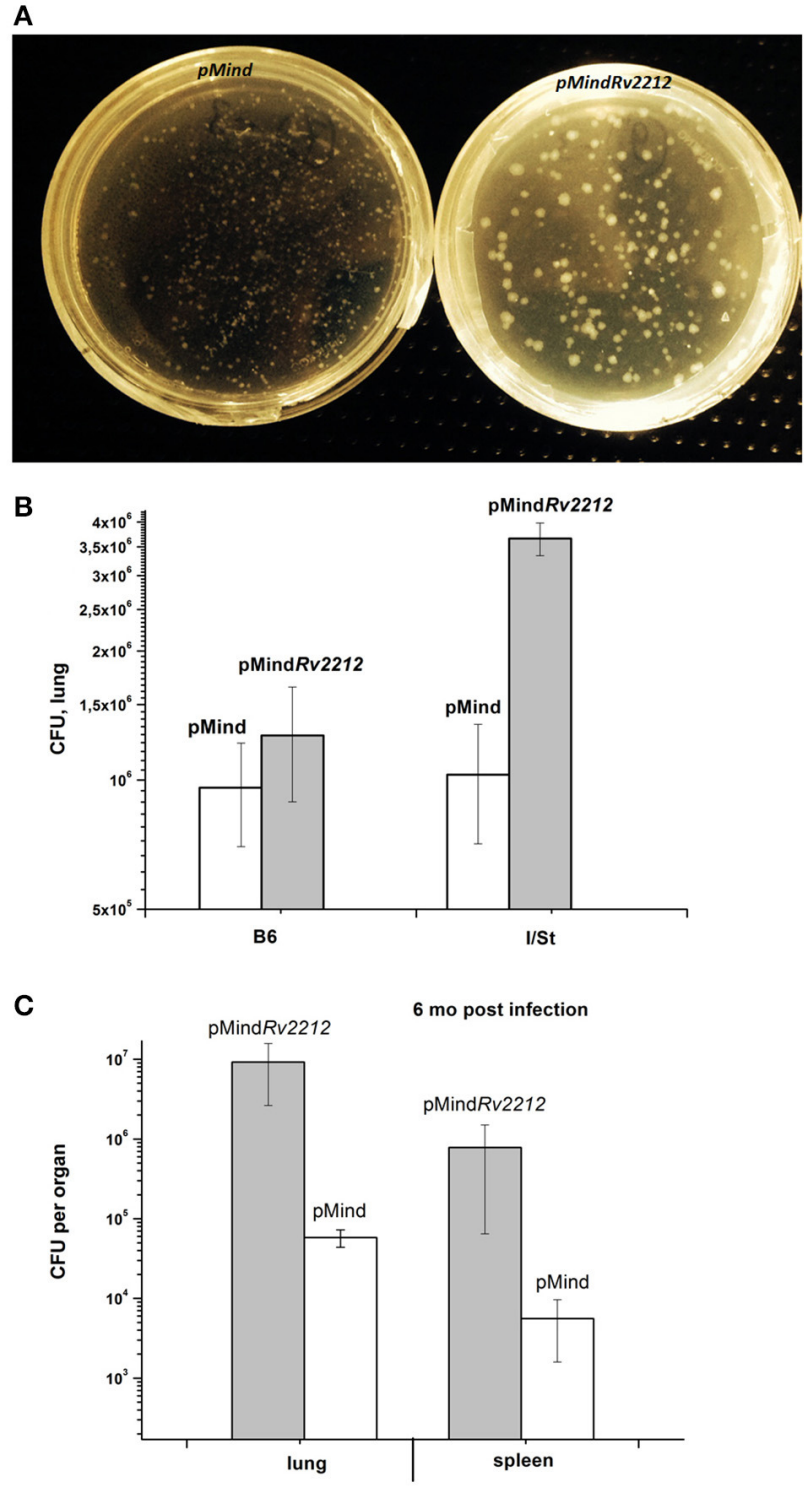
*M. tuberculosis* pMind*Rv2212* and pMind phenotypes expressed *in vivo*. **(A)** pMind*Rv2212* strain (left) isolated from lungs provided larger colonies compared to the control strain (right). Lungs of infected B6 mice were homogenized, plated on Dubos agar, kept at 37°C for 3 weeks and photographed. **(B)** Mice of I/St and B6 inbred strains were infected with 5 × 10^6^ CFUs of *M. tuberculosis* pMind*Rv2212* or pMind. At week three post-infection, lungs were homogenized and serial dilutions were plated on Dubo medium. The results of two experiments including 4 mice each (total *N* = 8) are expressed as mean ± SEM. Significant difference (*P* < 0.01, ANOVA) was observed between pMind*Rv2212*-infected B6 and I/St mice. **(C)** At 6 months post-challenge, ~2 log differences (*P* < 0.001, ANOVA) were observed between multiplication of pMind*Rv2212* and pMind mycobacteria in lungs and spleens of B6 mice. The results are expressed as mean ± SEM (*N* = 6).

**Figure 6:**
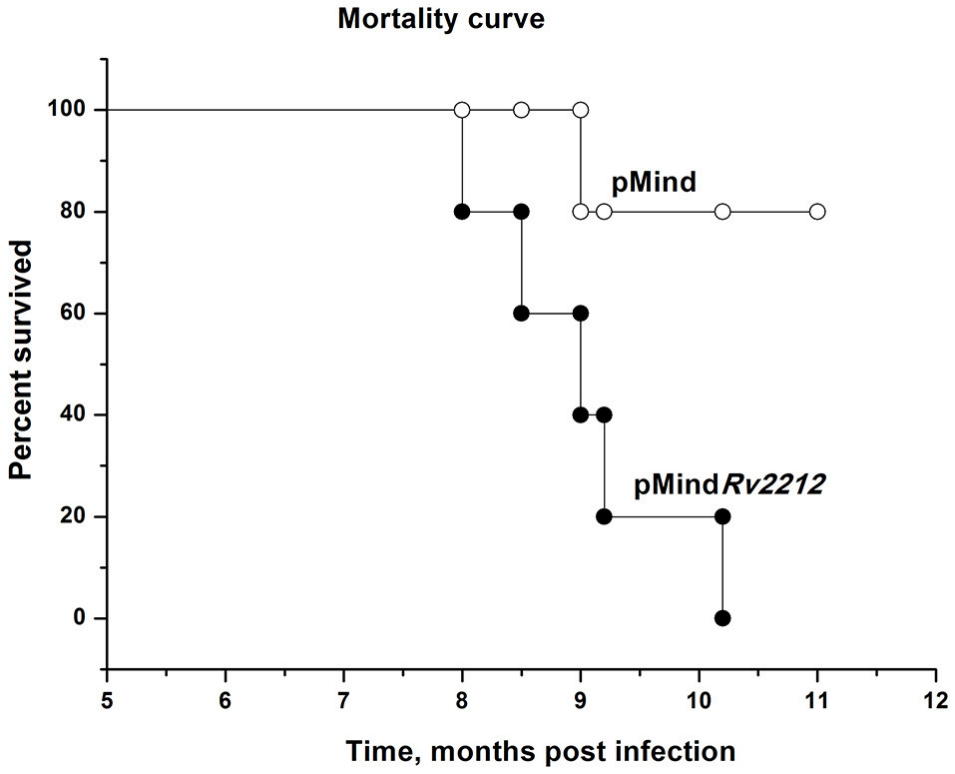
Mortality curve for *M. tuberculosis* pMind*Rv2212*- and pMind-infected B6 mice. Mice of B6 inbred strain were infected with 5 × 10^6^ CFUs of *M. tuberculosis* pMind*Rv2212* (closed circles) or pMind (open circles). Mortality was monitored weekly.

In previous experimentation, we and others characterized the phenotypes controlled by the *MSMEG_4279* and *Rv2212* genes *in vitro* and in cultured infected macrophages. In order to study the effect of *Rv2212* overexpression on the *bona fide* tuberculosis infection characteristics, mice of two inbred strains, genetically TB-resistant B6 and hyper-susceptible I/St (Nikonenko et al., 2000), were infected with pMind*Rv2212* and pMind bacteria. First, we obtained an indirect evidence for the AC expression contribution to mycobacterial vitality. Normally, in the absence of specific selection, mycobacteria eject any inserted plasmid if the coding gene does not provide any selection advantage. To assess the stability of the plasmid presence, at week 3 of infection lung homogenates were plated onto Dubo agar and the presence of the plasmid has been evaluated in the resulting bacterial colonies in the PCR format. Even in the absence of selecting hygromycin all colonies analyzed contained the plasmid with an intact Rv2212 insert, strongly suggesting that the AC overexpression is beneficial for the bacteria multiplying in the host. This conclusion was further supported by the observation that the pMind*Rv2212* colonies displayed markedly increased sizes compared to pMind colonies developed under identical conditions (Figure 5A). At the early stage of infection (3 weeks), the lung CFU counts in genetically susceptible I/St mice were significantly lower for the pMind strain compared to the pMind*Rv2212*, whereas genetically more resistant B6 mice controlled the infection caused by the two mycobacterial strains equally well (Figure 5B). However, at month 6 post-challenge, even B6 mice were unable to control the growth of pMind*Rv2212* strain in lungs, which was not the case for the pMind strain. Lung CFU counts increased ~1 log compared to the 3-week counts for the pMind*Rv2212* strain, and significant differences were also evident in spleens (Figure 5C). Importantly, in this experiment the CFU counts were identical for hygromycin-containing and antibiotic-free plates, confirming that the plasmid was retained by bacteria throughout the long, chronic infection course (data not shown). In agreement with the results of mycobacterial CFU evaluation, mortality curves obtained in B6 mice demonstrated that the pMind*Rv2212* strain was much more virulent in comparison with the control strain (Figure 6).

## Discussion

In this study, we present a few lines of evidence indicating the importance of AC expression and cAMP production for maintaining *Mtb* vitality under unfavorable for growth conditions. Firstly, there was a significant difference in *in vitro* growth dynamics between the AC-overexpressing pMind*Rv2212* and the control strain if the development of bacterial population started from a small amount of ancestors (Figure 1). Such “low inoculum” cultures are difficult to grow (Mukamolova et al., 2002; Drancourt and Raoult, 2007) and most often require additional external growth factors to initiate their active replication (Mukamolova et al., 2002). On the contrary, if a bulk inoculum is used for the initiation of mycobacterial liquid culture, the overexpression of the *Rv2212* gene does not influence growth dynamics (Figure S2). As shown earlier, *M. bovis* BCG with overexpressed *Rv2212* did not show a significant difference in growth *in vitro* compared to the wild type bacteria; however, only high initial cell concentrations were used in this study. Remarkably, this strain displayed a substantial growth acceleration compared to the control strain within mouse macrophages, i.e., when stressful conditions for intracellular parasite are evident (Pedroza-Roldán et al., 2015). Similarly, the overexpression of *Rv2212* in *M. smegmatis* resulted in an enhanced survival of bacteria within macrophages (Ganaie et al., 2016).

Secondly, the importance of cAMP production was confirmed in experiments on the development of dormant, NC bacteria *in vitro* under acidic stress. The AC overexpression resulted in the prolongation of bacterial capacity to form CFUs and in a significant delay or even prevention of bacterial transition toward dormancy (Figure 2). Thirdly, elevated *Rv2212* expression and cAMP production substantially stimulated the recovery of *M. tuberculosis* from the NC state and resuscitation of its active growth (Figure 4). Earlier we found that the NC *M. smegmatis* resuscitation was induced by exogenous fatty acids via *MSMEG_4279* (ortolog of *Rv2212*) activation followed by an increase in intracellular cAMP concentrations. The knocked-out *M. smegmatis* strain lacking *MSMEG_4279* was unable to resuscitate in the presence of fatty acids until exogenous cAMP was added (Shleeva et al., 2013). Finally, the *in vivo* experiments demonstrated that the increased level of AC expression (and evidently cAMP production) in the mutant stimulated *Mtb* multiplication in mouse organs.

This latter result is in a sharp contrast with observation by Pedroza-Roldán et al. (2015) concerning the *BCG-Rv2212* strain, which exhibited almost identical growth with control strain in mouse organs. We suspect that this difference may be connected with different locations of *Mtb* and *M. bovis* BCG within the host cells.

It was established that highly virulent mycobacterial species, e.g., *M. tuberculosis* and *M. leprae*, readily migrate from phagosomes into the cytosol of myeloid cells and continue their replication in this new cellular compartment (van der Wel et al., 2007). The fact that this migration is largely dependent upon the ESAT-6 protein, which perturbs the TLR2-MyD88 signaling pathway in the host cells, is significant for the prevention of mycobacterial migration from phagosomes (Rahman et al., 2014). In the genomes of the vast majority of non-virulent or low-virulent mycobacteria, including BCG, the secretory RD1 system, which contains genes for major virulence factors ESAT-6 and cfp10, is lacking (van Ingen et al., 2009), and a very large proportion of the total bacterial population indeed resides within macrophage phagosomes. Differences in intracellular cAMP concentrations might have different impact on the survival of mycobacteria residing within phagosome and cytosol compartments.

A relatively moderate increase in cAMP concentrations (~2.5-fold) appeared to be sufficient to elicit numerous growth-promoting effects of AC overexpression described above. The reason why such a subtle increase results in significant changes in bacterial physiology is unclear, given that during prolonged storage cAMP concentrations dropped 10-fold, whilst the numbers of NC bacteria showed ~5-log changes (Figure 3B). One possibility is that there exists a critical threshold for cAMP concentrations (18–20 pmol per 1 mg of wet weight), below which bacteria rapidly acquire NC phenotype, but even a moderate increase in the cAMP concentrations is sufficient for resuscitation and growth. Our earlier results demonstrating a rapid increase in cAMP content at the initial stage of *M. smegmatis* resuscitation support this conclusion (Shleeva et al., 2013).

There is ample evidence that cAMP production is essential for the survival of the pathogen within host cells. Thus, high cAMP amounts secreted by *Mtb* in macrophages modulate phagosome-lysosome fusion, thus increasing mycobacterial survival (Kalamidas et al., 2006). The macrophage “intoxication” by high cAMP concentrations may have a positive effect on mycobacterial survival (Agarwal et al., 2009). cAMP influences the expression of genes involved in mycobacterial response to hypoxic conditions often present *in vivo* (Gazdik and McDonough, 2005). High cAMP concentrations raise mycobacterial resistance to stressful conditions by elevating the expression of shock proteins GroEL and DnaK (Pedroza-Roldán et al., 2015). In addition, cAMP directly regulates the activity of such enzymes as malate dehydrogenase (Gazdik and McDonough, 2005) and lysine acetylase (Rv0998) (Knapp and McDonough, 2014) involved in the central mycobacterial metabolic pathways (Xu et al., 2011). These diverse effects can plausibly explain the better survival of the *Rv2212*-overexpressing strain *in vivo*.

Basing on the *in vitro* data, we suggest that a high level of cAMP interferes with bacterial transition to dormancy and development of latent-like disease *in vivo*. Further experiments are needed to elucidate this possibility, especially since the pMind*Rv2212* strain demonstrated an increased virulence in both genetically TB-susceptible and TB-resistant mice (Figures 5, 6). This is an important observation, suggesting that either mycobacterial AC itself or biochemical cascades upstream AC may be considered as a target for novel anti-TB drugs.

Also of note, that the molecular mechanisms increasing mycobacterial vitality under elevated cAMP concentrations remain obscure. The significance of intracellular cAMP as a second messenger for a wide variety of bacterial biochemical processes is well-established (Baker and Kelly, 2004), but the specific downstream processes linking cAMP levels and bacterial vitality on the transcriptional level are not well-defined. Two transcriptional factors sensing cAMP inside the cell were described: CRP_Mt_ (Rv3676) and Cmr (Rv1675c) (Knapp and McDonough, 2014). It was demonstrated that Cmr has more than 300 binding sites along a bacterial chromosome and thus potentially may be involved in regulating a wide variety of metabolic processes. In the context of present work, it is worth mentioning that cAMP modulates Cmr binding with the genes belonging to the DosR regulon, which is important for *Mtb* survival under hypoxic conditions in a non-replicating state (Ranganathan et al., 2016). CRP_Mt_ displays pleotropic effects as well, including the control of amino acid biosynthesis (Bai et al., 2011), expression of the *RpfA* gene encoding a mycobacterial resuscitation promoting factor (Mukamolova et al., 2003; Rickman et al., 2005; Shleeva et al., 2013), and the expression of succinate dehydrogenase (Rv0247c-Rv0249c) (Knapp et al., 2015).

Our study clearly demonstrates that elevated cAMP production due to the *Rv2212*-dependent AC activity plays an “anti-dormant” role by supporting, under unfavorable conditions, the active state of *Mtb* in culture and its multiplication inside the host. The exact metabolic structure of this “vitality supporting” axis remains to be identified.

## Author contributions

AK and MS conceived and designed the experiments. MS, GD, and AG performed the experiments *in vitro*. TK and ER performed the experiments *in vivo*. AK, MS, and AA analyzed the data. MS prepared figures and graphs. AK, MS, and AA wrote the manuscript. All the authors read and approved the final manuscript. AK, MS, and AA revised the manuscript.

### Conflict of interest statement

The authors declare that the research was conducted in the absence of any commercial or financial relationships that could be construed as a potential conflict of interest.

## Supplementary material

Figure S1 cAMP levels in different strains of *M. tuberculosis* grown on Sauton's medium for 8 days. Recombinant *M. tuberculosis* strains containing pMind*Rv2212* vector or empty pMind vector were cultured in the standard Sauton's medium with agitation (200 rpm) at 37°C for 8 days. In some experiments, tetracycline hydrochloride was added to medium at the final concentration 20 ng per ml. Results display the average from three independent experiments.

Figure S2 Growth of different *M. tuberculosis* strains in the standard Sauton's medium from the 10^5^ inoculum. Recombinant *M. tuberculosis* strains containing pMind*Rv2212* vector (closed circles) or empty pMind vector (open circles) were cultured in the standard Sauton's medium with agitation (200 rpm) at 37°C. Initial size of inoculum was 10^5^ bacteria per ml. This experiment was repeated 3 times with similar results, one representative experiment is shown.

## References

[B1] Abdel Motaal A. Tews I. Schultz J. E. Linder J. U. (2006). Fatty acid regulation of adenylyl cyclase Rv2212 from *Mycobacterium tuberculosis* H37Rv. FEBS J. 273, 4219–4228. 10.1111/j.1742-4658.2006.05420.x 16925585

[B2] Agarwal N. Lamichhane G. Gupta R. Nolan S. Bishai W. R. (2009). Cyclic AMP intoxication of macrophages by a *Mycobacterium tuberculosis* adenylate cyclase. Nature 460, 98–102. 10.1038/nature08123 19516256

[B3] Bai G. Schaak D. D. Smith E. A. McDonough K. A. (2011). Dysregulation of serine biosynthesis contributes to the growth defect of a *Mycobacterium tuberculosis* crp mutant. Mol. Microbiol. 82, 180–198. 10.1111/j.1365-2958.2011.07806.x 21902733 PMC3785234

[B4] Baker D. A. Kelly J. M. (2004). Structure, function and evolution of microbial adenylyl and guanylyl cyclases. Mol. Microbiol. 52, 1229–1242. 10.1111/j.1365-2958.2004.04067.x 15165228

[B5] Barry C. E. III. Boshoff H. I. Dartois V. Dick T. Ehrt S. Flynn J.. (2009). The spectrum of latent tuberculosis: rethinking the biology and intervention strategies. Nat. Rev. Microbiol. 7, 845–855. 10.1038/nrmicro2236 19855401 PMC4144869

[B6] Connell N. D. (1994). *Mycobacterium*: isolation, maintenance, transformation, and mutant selection. Methods Cell Biol. 45, 107–125. 7707982 10.1016/s0091-679x(08)61848-8

[B7] de Man J. C. (1975). The probability of most probable numbers. Eur. J. Appl. Microbiol. 1, 67–78.

[B8] Drancourt M. Raoult D. (2007). Cost-Effectiveness of Blood Agar for Isolation of Mycobacteria. PLoS Negl. Trop. Dis. 1:e83. 10.1371/journal.pntd.0000083 18060087 PMC2100370

[B9] Ganaie A. A. Trivedi G. Kaur A. Jha S. S. Anand S. Rana V.. (2016). Interaction of Erp protein of *Mycobacterium tuberculosis* with Rv2212 enhances intracellular survival of *Mycobacterium smegmatis*. J. Bacteriol. 198, 2841–2852. 10.1128/JB.00120-16 27481930 PMC5038003

[B10] Gazdik M. A. McDonough K. A. (2005). Identification of cyclic AMP-regulated genes in *Mycobacterium tuberculosis* complex bacteria under low-oxygen conditions. J. Bacteriol. 187, 2681–2692. 10.1128/JB.187.8.2681-2692.2005 15805514 PMC1070381

[B11] Kalamidas S. A. Kuehnel M. P. Peyron P. Rybin V. Rauch S. Kotoulas O. B.. (2006). cAMP synthesis and degradation by phagosomes regulate actin assembly and fusion events: consequences for mycobacteria. J. Cell Sci. 119, 3686–3694. 10.1242/jcs.03091 16931599

[B12] Knapp G. S. McDonough K. A. (2014). Cyclic AMP signaling in Mycobacteria. Microbiol. Spectr. 2, MGM2-0011-2013. 10.1128/microbiolspec.MGM2-0011-2013 26105822

[B13] Knapp G. S. Lyubetskaya A. Peterson M. W. Gomes A. L. Ma Z. Galagan J. E.. (2015). Role of intragenic binding of cAMP responsive protein (CRP) in regulation of the succinate dehydrogenase genes Rv0249c-Rv0247c in TB complex mycobacteria. Nucleic Acids Res. 43, 5377–5393. 10.1093/nar/gkv420 25940627 PMC4477654

[B14] Mukamolova G. V. Kaprelyants A. S. Kell D. B. Young M. (2003). Adoption of the transiently non-culturable state–a bacterial survival strategy? Adv. Microb. Physiol. 47, 65–129. 10.1016/S0065-2911(03)47002-1 14560663

[B15] Mukamolova G. V. Turapov O. A. Young D. I. Kaprelyants A. S. Kell D. B. Young M. (2002). A family of autocrine growth factors in *Mycobacterium tuberculosis*. Mol. Microbiol. 46, 623–635. 10.1046/j.1365-2958.2002.03184.x 12410821

[B16] Nikonenko B. V. Averbakh M. M. Jr. Lavebratt C. Schurr E. Apt A. S. (2000). Comparative analysis of mycobacterial infections in susceptible I/St and resistant A/Sn inbred mice. Tuber. Lung Dis. 80, 15–25. 10.1054/tuld.1999.0225 10897380

[B17] Pedroza-Roldán C. Aceves-Sánchez M. J. Zaveri A. Charles-Niño C. Elizondo-Quiroga D. E. Hernández-Gutiérrez R.. (2015). The adenylyl cyclase Rv2212 modifies the proteome and infectivity of *Mycobacterium bovis* BCG. Folia Microbiol. 60, 21–31. 10.1007/s12223-014-0335-1 25038956

[B18] Rahman V. F. Sobia P. Gupta N. Kaer L. V. Das G. (2014). *Mycobacterium tuberculosis* subverts the TLR-2 – MyD88 pathway to facilitate its translocation into the cytosol. PLoS ONE 9:e86886. 10.1371/journal.pone.0086886 24475192 PMC3903598

[B19] Ranganathan S. Bai G. Lyubetskaya A. Knapp G. S. Peterson M. W. Gazdik M.. (2016). Characterization of a cAMP responsive transcription factor, Cmr (Rv1675c), in TB complex mycobacteria reveals overlap with the DosR (DevR) dormancy regulon. Nucleic Acids Res. 44, 134–151. 10.1093/nar/gkv889 26358810 PMC4705688

[B20] Rickman L. Scott C. Hunt D. M. Hutchinson T. Menéndez M. C. Walan R.. (2005). A member of the cAMP receptor protein family of transcription regulators in Mycobacterium tuberculosis is required for virulence in mice and controls transcription of the rpfA gene coding for a resuscitation promoting factor. Mol. Microbiol. 56, 1274–1286. 10.1111/j.1365-2958.2005.04609.x 15882420 PMC2964915

[B21] Shleeva M. O. Bagramyan K. Telkov M. V. Mukamolova G. V. Young M. Kell D. B.. (2002). Formation and resuscitation of “non-culturable” cells of *Rhodococcus rhodochrous* and *Mycobacterium tuberculosis* in prolonged stationary phase. Microbiology 148, 1581–1591. 10.1099/00221287-148-5-1581 11988533

[B22] Shleeva M. O. Kudykina Y. K. Vostroknutova G. N. Suzina N. E. Mulyukin A. L. Kaprelyants A. S. (2011). Dormant ovoid cells of *Mycobacterium tuberculosis* are formed in response to gradual external acidification. Tuberculosis 91, 146–154. 10.1016/j.tube.2010.12.006 21262587

[B23] Shleeva M. Goncharenko A. Kudykina Y. Young D. Young M. Kaprelyants A. (2013). Cyclic AMP-dependent resuscitation of dormant Mycobacteria by exogenous free fatty acids. PLoS ONE 8:e82914. 10.1371/journal.pone.0082914 24376605 PMC3871856

[B24] van der Wel N. Hava D. Houben D. Fluitsma D. van Zon M. Pierson J. (2007). *M. tuberculosis* and *M. leprae* translocate from the phagosome to the cytosol in myeloid cells. Cell 129, 1287–1298. 10.1016/j.cell.2007.05.059 17604718

[B25] van Ingen J. Zwa R. Dekhuijzen R. Boeree M. van Soolingen D. (2009). Region of Difference 1 in nontuberculous *Mycobacterium* species adds a phylogenetic and taxonomical character. J. Bacteriol. 191, 5865–5867. 10.1128/JB.00683-09 19617365 PMC2737943

[B26] Xu H. Hegde S. S. Blanchard J. S. (2011). Reversible acetylation and inactivation of *Mycobacterium tuberculosis* acetyl-CoA synthetase is dependent on cAMP. Biochemistry 50, 5883–5892. 10.1021/bi200156t 21627103 PMC3125470

