# Supplementary figures and images for: Overexpression of Adenylyl Cyclase Encoded by the Mycobacterium tuberculosis Rv2212 Gene Confers Improved Fitness, Accelerated Recovery from Dormancy and Enhanced Virulence in Mice
Source: Front Cell Infect Microbiol. 2017 Aug 17;7:370. doi: 10.3389/fcimb.2017.00370 (PMC5562752; doi:10.3389/fcimb.2017.00370)

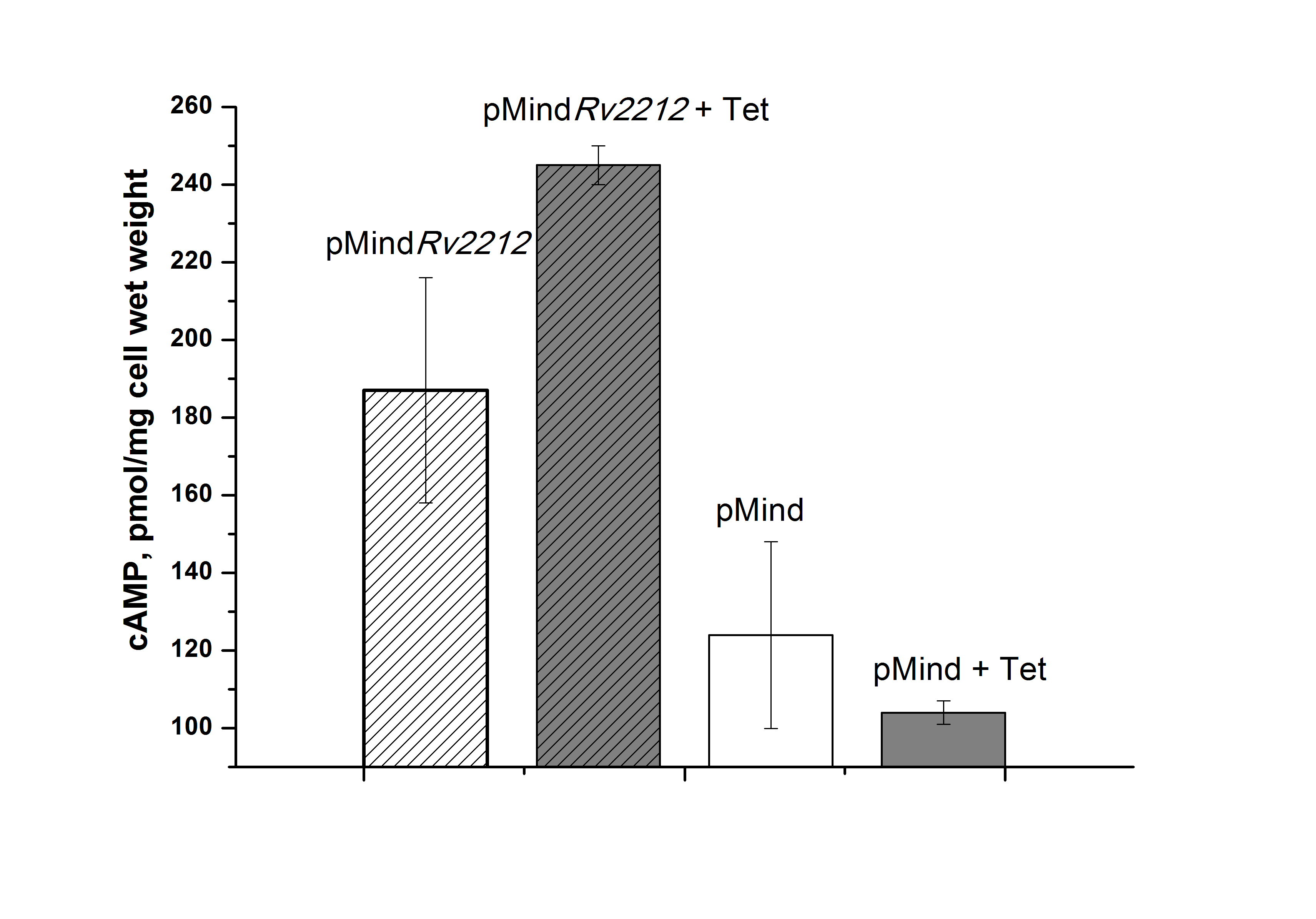

Supplement: Figure S1 — cAMP levels in different strains of M. tuberculosis grown on Sauton's medium for 8 days. Recombinant M. tuberculosis strains containing pMindRv2212 vector or empty pMind vector were cultured in the standard Sauton's medium with agitation (200 rpm) at 37°C for 8 days. In some experiments, tetracycline hydrochloride was added to medium at the final concentration 20 ng per ml. Results display the average from three independent experiments. [file Image1.JPEG]

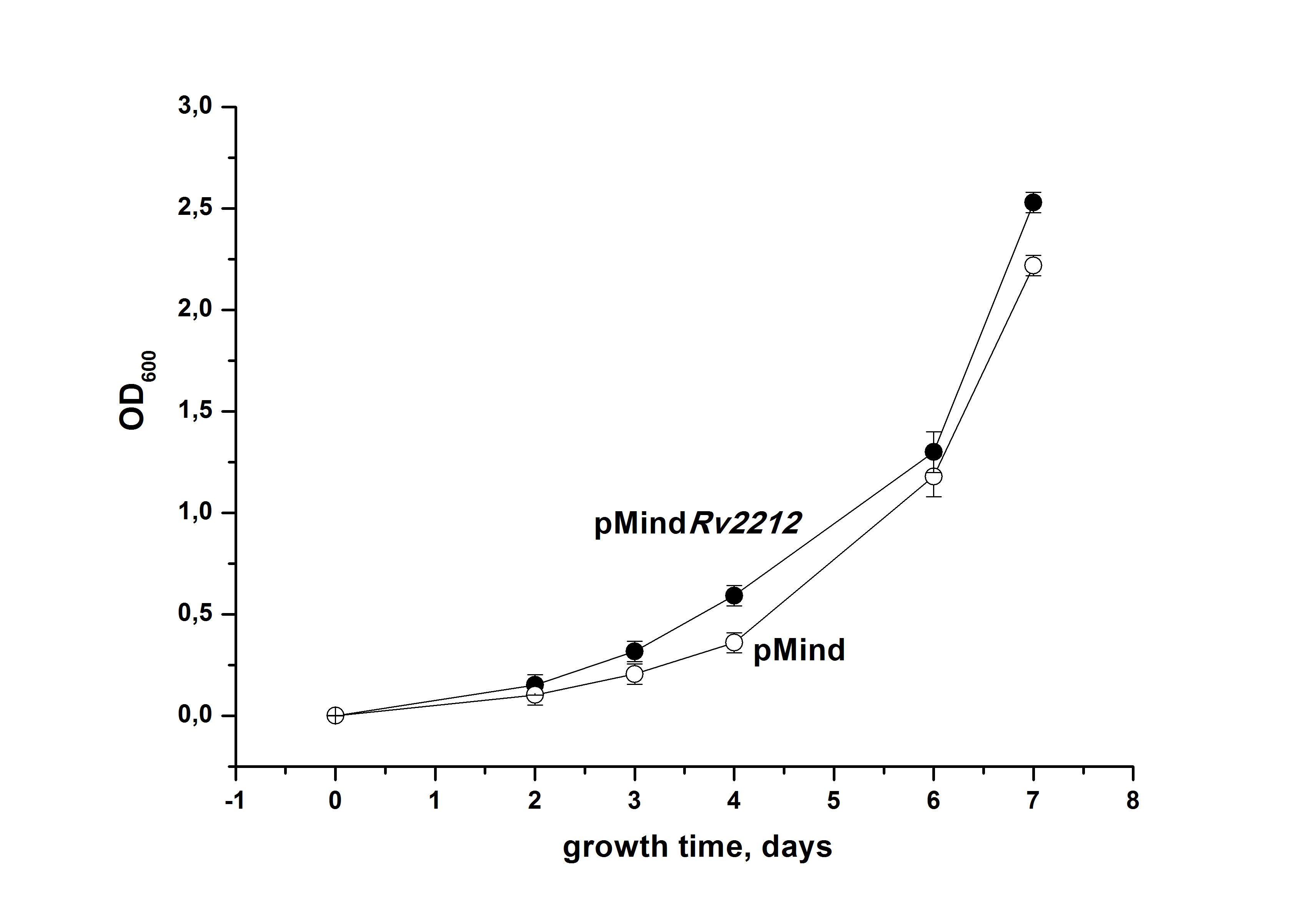

Supplement: Figure S2 — Growth of different M. tuberculosis strains in the standard Sauton's medium from the 105 inoculum. Recombinant M. tuberculosis strains containing pMindRv2212 vector (closed circles) or empty pMind vector (open circles) were cultured in the standard Sauton's medium with agitation (200 rpm) at 37°C. Initial size of inoculum was 105 bacteria per ml. This experiment was repeated 3 times with similar results, one representative experiment is shown. [file Image2.JPEG]
